# Acetylcholinesterase Inhibition-Based Biosensor for Aluminum(III) Chronoamperometric Determination in Aqueous Media

**DOI:** 10.3390/s140508203

**Published:** 2014-05-07

**Authors:** Miriam Barquero-Quirós, Olga Domínguez-Renedo, Maria Asunción Alonso-Lomillo, María Julia Arcos-Martínez

**Affiliations:** 1 University of Costa Rica, CELEQ, San Pedro de Montes de Oca, 11500-2060 San José, Costa Rica; 2 Department of Chemistry, Faculty of Sciences, University of Burgos, Plaza Misael Bañuelos s/n, 09001 Burgos, Spain; E-Mails: olgado@ubu.es (O.D.-R.); malomillo@ubu.es (M.A.A.-L.); jarcos@ubu.es (M.J.A.-M.)

**Keywords:** acetylcholinesterase, biosensor, aluminum, acetylthiocholine iodide, screen-printed electrodes, gold nanoparticles

## Abstract

A novel amperometric biosensor for the determination of Al(III) based on the inhibition of the enzyme acetylcholinesterase has been developed. The immobilization of the enzyme was performed on screen-printed carbon electrodes modified with gold nanoparticles. The oxidation signal of acetylthiocholine iodide enzyme substrate was affected by the presence of Al(III) ions leading to a decrease in the amperometric current. The developed system has a detection limit of 2.1 ± 0.1 μM for Al(III). The reproducibility of the method is 8.1% (n = 4). Main interferences include Mo(VI), W(VI) and Hg(II) ions. The developed method was successfully applied to the determination of Al(III) in spiked tap water. The analysis of a certified standard reference material was also carried out. Both results agree with the certified values considering the respective associated uncertainties.

## 1. Introduction

Aluminum determination at trace level is of great importance due to the possible human and environmental toxic effects of this element. In fact, it has been demonstrated that low levels of aluminum in human organism, are related with Alzheimer disease [1–3].

Traditionally, the determination of aluminum at low concentration levels in environmental and biological samples, has been carried out by means of electrothermal absorption spectrometry [4–6] and fluorescence spectroscopy [7]. However these methods have often failed in routine analysis. Electrochemical techniques, such as voltammetry, have been also employed using different kinds of electrodes, being the hanging mercury drop electrode (HMDE) the most often used one in the analysis of Al(III). The direct voltammetric determination of Al(III) with a HMDE is limited by the difficulty of reduction of Al(III) species and the concurrent reduction of hydrogen ions from aqueous acid solution at mercury electrodes. This difficulty is usually overcome by complexation of Al(III) with many different chelating agents, which electrochemical reduction takes place at lower potentials than free Al(III) ions and other electrochemical techniques [8–30]. Despite of the good results obtained with mercury electrodes, the use of HMDE is nowadays being reduced due to its environmental pollution problems.

Screen-printed electrodes (SPEs) have demonstrated numerous advantages as transducers in electrochemical biosensors [31]. These advantages include ease of modification with different compounds such as nanoparticles (NPs) in order to create a more favorable electrode surface for interaction with enzymes. This kind of modification generally produces a more stable biosensor for the determination of different analytes [32,33]. Therefore, nowadays the use of NPs as surface electrode modificators is increasing showing these modified electrodes great electrocatalytic activity and therefore high analytical response [34]. Actually, screen-printed modified electrodes with enzymes and gold NPs (AuNPs) are foreseeing as selective and sensitive methods to analytical quantification of a broad variety of environmental contaminants [35].

The determination of aluminum has been also carried using an enzymatic biosensor based on the inhibitory effect of this element on the enzyme α-chymotrypsin [36]. In this way, the fact, that the acetylcholinesterase (AChE) enzyme is also usually inhibited by different compounds such as organophosphorate pesticides, metals and nonmetals [37–40] has led us to develop a new AChE based biosensor for the analysis of aluminum.

In this work, screen-printed carbon electrodes (SPCEs) were modified with AuNPs (AuNPs/SPCEs). AChE was immobilized on the surface of the developed AuNPs/SPCEs. The chronoamperometric response of these AChE/AuNPS/SPCE biosensors towards acetylthiocholine (ATI) enzyme substrate was affected by the presence of Al(III) ions due to their inhibitor effect on the activity of the enzyme. To the best of our knowledge, there are no references about other biosensors using this enzyme for the determination of Al(III).

## 2. Results and Discussion

The AChE/AuNPS/SPCE biosensors developed in this work show a sensitive chronoamperometric response to the enzyme substrate, ATI. This signal is considerably affected by the presence of Al(III) ions that produce a decrease in the recorded chronoamperometric response. This inhibition effect can be quantitatively related to the concentration of Al(III).

The chronoamperometric response of the developed biosensors is affected by different experimental variables namely, operational chronoamperometric potential, pH and concentration of enzyme substrate (acetylthiocholine iodide). Therefore, an optimization procedure of these experimental parameters was carried out.

The electrochemical response of acetylthiocholine iodide was evaluated first, through a surface response method using a 2^3^ composite central experimental design. In this case, three parameters were studied in order to maximize the current registered, ΔI (I0-I), for a 1.4 μM aluminum solution: applied potential (from 0.63 V to 0.97 V, *vs.* screen-printed Ag/AgCl electrode), pH of supporting electrolyte (from 6.96 to 8.64) and substrate concentration (from 0.038 mM to 0.44 mM). The experiments carried out in different experimental conditions led to establishment of the following optimum values: pH = 8.6; ATI = 0.44 mM; Eap = 0.76 V, but the calibration curves obtained under these conditions were too noisy. Those were improved with the conditions obtained from the second study where it was done a sequential optimization; choosing the conditions suggested by next figures and regarding also enzyme activity.

**Figure 1.**
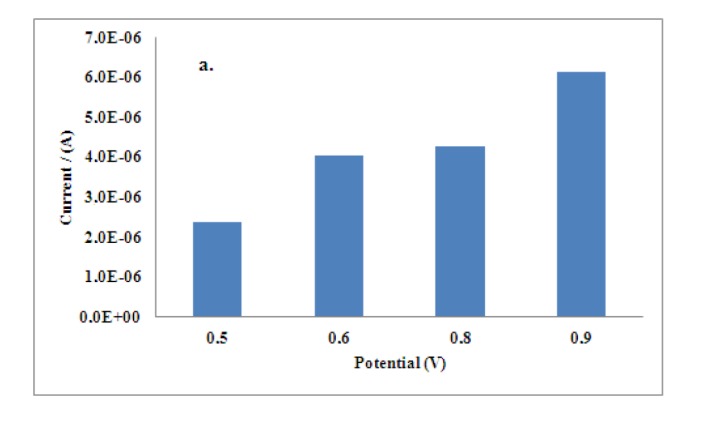

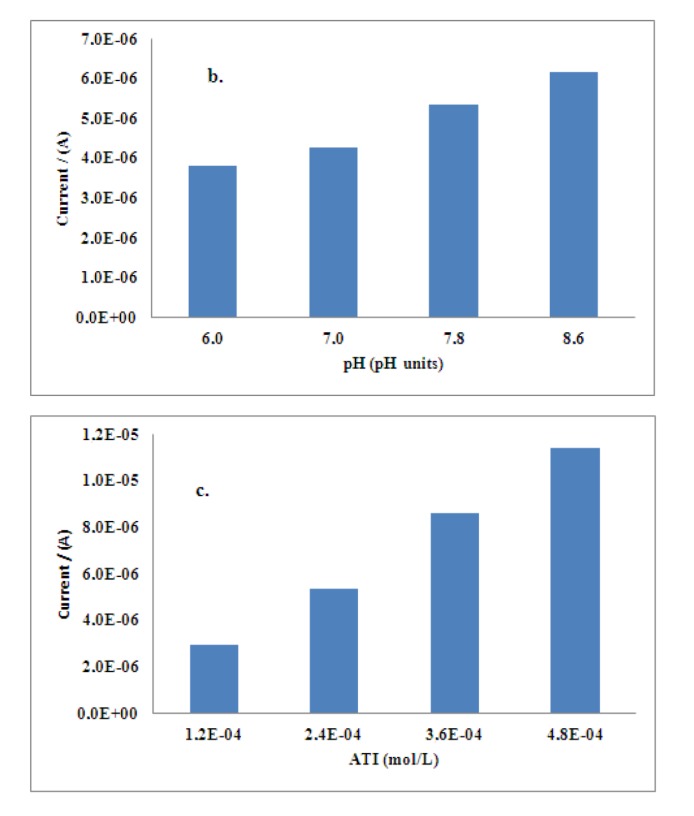
(**a**) Current response of ATI with potential, (**b**) Current response of ATI with pH, (**c**) Current response of ATI concentration.

First of all, the effect of applied potential in the chronoamperometric response of the developed biosensors was studied. The inhibitive signal of Al(III) on the disposable electrodes was analyzed using operational potentials from + 0.5 to + 0.9 V at pH 7.0, recommended for enzyme. A high quality amperometric signal at + 0.8 V was obtained, thus, this potential was taken as the best value for determination of aluminum. The influence of pH value was also studied. Chronoamperometric analysis in a pH range from 6 to 9 was performed obtaining a value of 7.8 as the optimum taking into account the best stability conditions for the enzymatic electrode. Finally, the influence of the concentration of acetylthiocholine iodide was also studied in the range from 0.12 mM up to 0.48 mM, a concentration of 0.24 mM was found to give the highest inhibition response of aluminum, upper concentrations were noisy. The results are showed in Figure 1.

**Figure 2.**
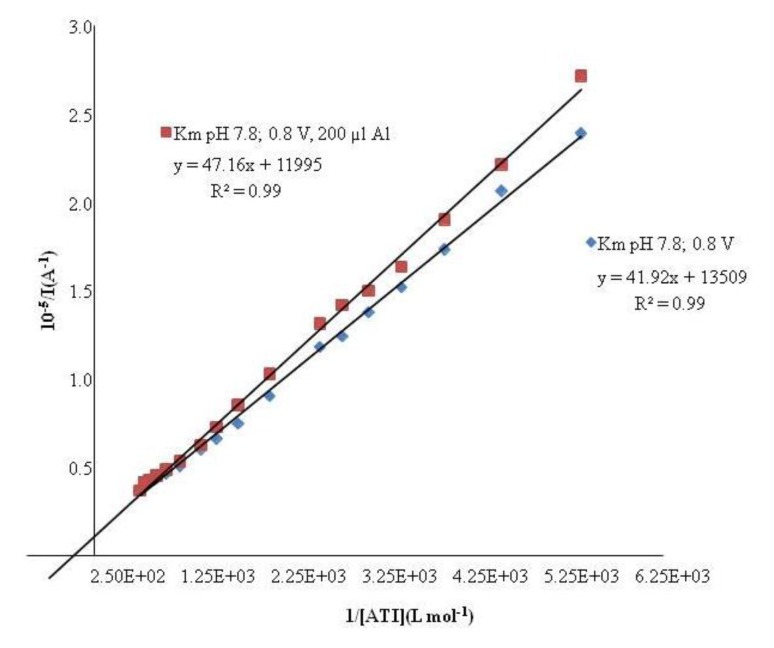
Lineweaver Burk plot of AChE/AuNPs/SPCE biosensor in presence of aluminum and without aluminum.

Once the optimal conditions were obtained, the inhibitory effect of aluminum ions on the response of the AChE biosensors was investigated following the method of Lineweaver-Burk. The Km value (3.9 ± 0.3) × 10^−3^ M found in presence of aluminum is higher than the one obtained without aluminum (3.1 ± 0.3) × 10^−3^. Therefore, the presence of aluminum diminishes the enzyme substrate affinity. In Figure 2 it can be seen that the studied process resembles a non competitive inhibition.

### 2.1. Calibration and Limit of Detection

**Figure 3.**
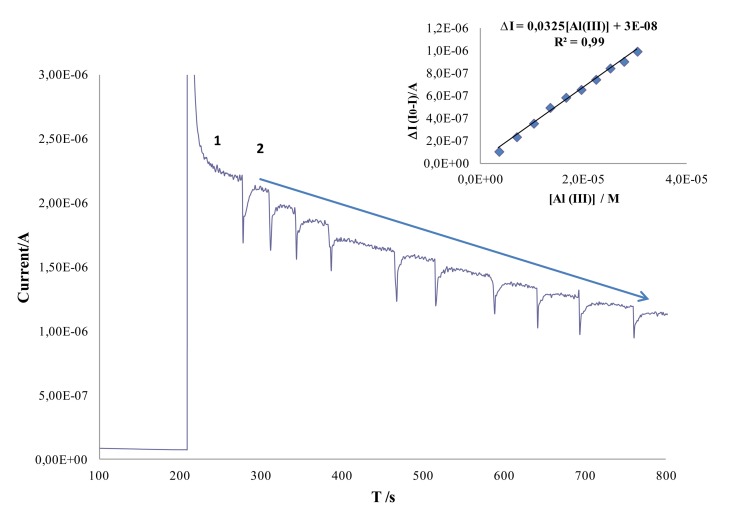
Typical amperometric recording for an acethylthiocholine iodide concentration (1) 2.4 × 10^−4^ M and consecutive additions of aliquots of Al(III) solution into the cell to give an overall concentration of: (2) 3.6 × 10^−6^, (3) 7.0 × 10^−6^, (4) 1.0 × 10^−5^, (5) 1.3 × 10^−5^, (6) 1.6 × 10^−5^, (7) 1.9 × 10^−5^, (8) 2.2 × 10^−5^, (9) 2.5 × 10^−5^, (10) 2.8 × 10^−5^, (11) 3.0 × 10^−5^; Britton-Robinson pH 7.8; Eap, + 0.8 V *vs.* Ag/AgCl screen-printed electrode.

Figure 3 shows a typical chronoamperometric response obtained using AChE/AuNPs/SPCEs.

As it can be seen in this figure, biosensor responds fast to aluminum concentration and a linear dependence between the decrease (ΔI) in the chronoamperometric response of ATI (I0) and the Al(III) concentration (I) was observed in the concentration range from 3.6 μM to 30 μM. The regression parameters obtained for the calibration curve showed in Figure 3 were ΔI = 0.0325 [Al(III)] + 3.0 × 10^−8^ (R^2^ = 0.99).

Several calibration curves were constructed under the optimum conditions in order to evaluate figures of merit, such as precision and limit of detection. The limit of detection under the optimum working conditions (2.1 ± 0.1) μM was calculated from the standard deviation of seven calibration curves (Sy/x) accordingly with the criteria 3Sy/x.

### 2.2. Precision

This parameter was calculated in terms of reproducibility. Repeatibility was tried out using the same electrode surface. In this way, several successive calibrations for Al(III) were tested. The electrodes were conditioned for 5 min in a stirred Britton Robinson buffer solution, pH 7, between experiments. The relative standard deviation (RSD) obtained for the slopes of the first two curves was lower than 4%, but from the third measurement an increase in RSD was noticed. Likewise, the reproducibility of the amperometric signal was checked using the slopes of four regressions carried out with different electrode surfaces. The RSD value obtained was 8.1%. These results suggest that the fabrication procedure of the AChE/AuNPs/SPCEs biosensors is reliable and allows reproducible electroanalytical responses to be obtained with different electrodes constructed using the method described in this work.

### 2.3. Accuracy

The accuracy of the developed method was evaluated by means of the analysis of a standard reference material (SRM) High Purity Standards solution (Lot Number 1121015, (1000 ± 3) mg/L) using the standard addition method. The aluminum average concentration quantified by the developed procedure, (1022 ± 20) mg/L (n = 4; α= 0.05), matches the certified value of the sample considering the associated uncertainty. The mean value percent recovery obtained was 102 ± 2.

The performance of the method was also evaluated by means of the analysis of spiked tap water samples. These samples were prepared by the addition of different amounts of the above described standard reference material. Accordingly with the results obtained for this analysis the average value found, (1058 ± 63) mg/L (n = 4; α = 0.05), is in good agreement with the certified value of the SRM. The average value percent recovery was 106 ± 8. These results suggest that the developed method with acetylcholinesterase enzyme is accurate and reliable for aluminum determination in water.

### 2.4. Interferences

**Figure 4.**
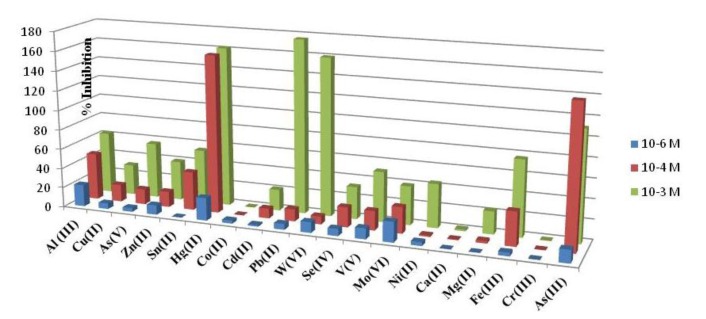
Inhibition percentage of inhibition current of ACh AuNPs/SPCE in presence of: Cu(II); As(V); As(III); Zn(II); Sn(II); Hg(II); Co(II); Cd(II); Pb(II); W(VI); Se(IV); V(V); Mo(VI); Ni(II);Ca(II); Mg(II); Fe(III); Cr(III); As(III) at three levels of concentration; [ATI] 0.12 μM; Eap, + 0.8 V *vs.* Ag/AgCl screen printed electrode; Britton-Robinson pH 7.8.

**Figure 5.**
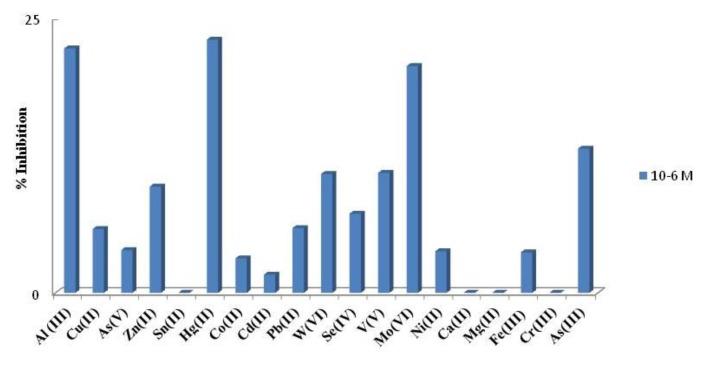
Inhibition percentage of inhibition current of ACh AuNPs/SPCE in presence of: Cu(II); As(V); As(III); Zn(II); Sn(II); Hg(II); Co(II); Cd(II); Pb(II); W(VI); Se(IV); V(V); Mo(VI); Ni(II);Ca(II); Mg(II); Fe(III); Cr(III); As(III) at 10^−6^ M; [ATI] 0.12 μM; Eap, + 0.8 V *vs.* Ag/AgCl screen printed electrode; Britton-Robinson pH 7.8.

An interference study was performed comparing the percentage of inhibition, showed for the developed acetylcholinesterase-based biosensor in the presence of aluminum and others foreign ions. Three concentration levels were tested, namely 1 mM; 0.1 mM and 1 μM. As it can be seen in Figure 4 the highest interference effect was found for Mo(VI), W(VI) and Hg(II). The interference of As(III) was performed by mixing 1000 μL of standard solution of As(V) 1.33 × 10^−2^ M with 1,000 μL of sodium thiosulfate 0.1 M and adding the necessary amount to reach level concentration tested. The mixture was left to react for 70 min at room and used immediately. Under the aluminum biosensor conditions although As(III) is an important interference is not the strongest, considering that the most stable specie in aqueous solutions is As (V). It is important to point out that calcium and magnesium showed a low interference at any level of concentration as it can be seen in Figure 4. Moreover, from Figure 5 it can be also deduced that when Al(III) is presented at low concentrations (1 μM), Hg (II), As (III) and Mo(VI) are interferences at the same low concentration level.

## 3. Reagents and Equipment

### 3.1. Reagents

Several inks were used in the fabrication of SPEs, namely Electrodag PF-407 A (carbon ink), Electrodag 6037 SS (silver/silver chloride ink) and Electrodag 452 SS (dielectric ink) supplied by Acheson Colloiden (Scheemda, The Netherlands).

Hand-made SPEs were produced on a DEK 248 printing machine (DEK, Weymouth, UK) using polyester screens with appropriate stencil designs mounted at 45° to the printer stroke.

All solutions were prepared with purified water supplied by TKA Purification System, inverse osmosis, with a UV lamp irradiation system.

AChE enzyme (200–1000 U/mg) and N-cyclohexyl-N-2-morpholinoethylcarbodiimide methyl *p*-toluene sulfonate were purchased from Sigma (Steinheim, Germany) and ATI was purchased from Fluka (Buchs, Switerzland). Bovine serum albumine (BSA) and hydrogen tetrachloroaurate (III) trihydrate (HAuCl_4_) were obtained from Sigma-Aldrich (Sigma-Aldrich, Steinheim, Germany).

Stock standard solutions of Al, Fe, Cu, Sn, Zn, Co, Ni, Se Cr, Cd, Pb and Se were prepared from Titrisol solutions (Merck, Darmstad, Germany). Solutions of V, Mo, W and Mg were acquired from High Purity Standard (Charleston, SC, USA). Ca solution used was obtained from Inorganic Ventures Lakewood (Lakewood, NJ, USA). As and Hg solutions were prepared from Atomic Spectroscopy Standards solutions (Perkin-Elmer Co., Norwalk, CT, USA).

Britton Robinson supporting electrolyte solutions were prepared as usual with boric, phosphoric and acetic acids (Merck). All pH values were obtained adjusting with a NaOH solution (Suprapur, Merck, Darmstadt, Germany).

Al(III) solution used in the analysis of spiked water samples was purchased from High Purity Standard confirmed against standard reference material SRM 3101.

### 3.2. Biosensor Manufacturing

An electrochemical system Autolab PGSTAT Echochemie128 N with GPS software was used to record electrochemical measurements (Echochemie, Utrech, Netherlands). All necessary pH values were adjusted with a pHmeter (Mettler Toledo, Schwerzenbach, Suitzerland).

### 3.3. Screen-Printed Electrodes Preparation

**Figure 6.**
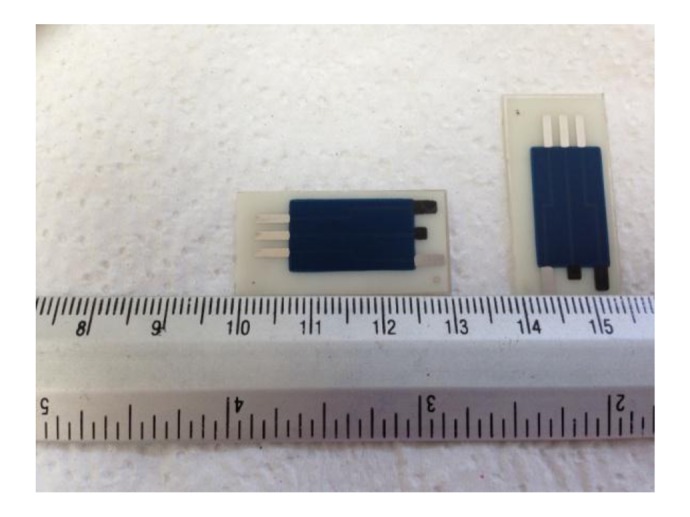
Dimensions of the screen printed electrode system used.

The construction of the hand-made SPEs used in the determination of aluminum was based on printing successive layers of different inks onto a polyester strip substrate. Four different screens with appropriate stencils were used to transfer the required design following the printing procedure described in previous works [41]. A picture of dimensions of the SPE used is showed in Figure 6.

### 3.4. Modification of Screen-Printed Carbon Electrodes With Gold Nanoparticles

Metallic AuNPs deposits were obtained by direct electrochemical deposition on the screen-printed carbon electrode (SPCE) surface using a 0.1 mM solution of HAuCl_4_ in 0.5 M H_2_SO_4_. The deposition was performed by applying a potential of + 0.18 V during 15 s under stirring conditions [42].

### 3.5. Acetyilcholinesterase Enzyme Inmobilization on Gold Nanoparticles Modified Screen-Printed Carbon Electrode

The enzyme was immobilized by covalent union using N-cyclohexyl-N'-2-morpholino-ethylcarbodiimide methyl *p*-toluene sulfonate, on the surface of AuNPs/SPCEs [43]. A 0.07 M solution of N-cyclohexyl-N-2-morpholinoethylcarbodiimide methyl *p*-toluene sulfonate was prepared in Britton Robinson pH 7.0. Aliquots of this solution were placed in vials of 100 μL and stored frozen; one vial was thawed each time that it was necessary to immobilize the enzyme over the AuNPs/SPCE surface. To carry out the immobilization procedure 5 μL of the buffer solution with cyclohexyl-N-2- morpholinoethylcarbodiimide methyl *p*-toluene sulfonate were placed on the working electrode surface, and an activation period of 80 min was elapsed before the next step that consisted of addition of enzyme.

**Figure 7.**
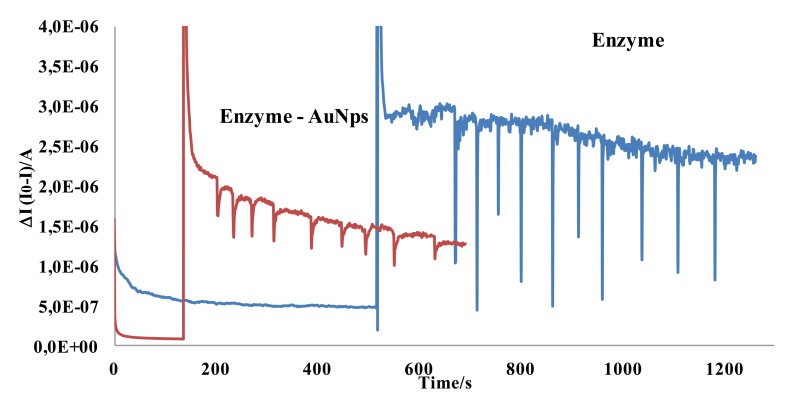
Calibration curves realized with enzyme acetilcholinesterase and AuNps and without AuNps.

An acetylcholinesterase solution was prepared by dissolving 300 mg of the enzyme in 1 mL of Britton Robinson pH 7.0 solution. Aliquots of this enzyme solution were placed in vials and stored frozen; one vial was thawed each time. Next, 5 μL of this enzyme solution were placed onto the working electrode surface and left to react at 30 °C for two hours. The electrode was finally stored at 4 °C. The modified electrode was washed with a pH 7 buffer solution, before using and between measurements. The modification of a SPCE with enzymes leads to an important change in the electrical double layer that produces a decrease in the rate of electron transfer [44–46], and the inhibitory effect is perfectly measurable with this type of biosensors. The performance of SPCE modified with AuNPs has been widely showed [47–49]. In fact, experiments carried out without gold nanoparticles lead to poor results as can be seen in Figure 7.

### 3.6. Chronoamperometric Determination of Aluminium

The AChE/AuNPs/SPCE biosensor was placed in an electrochemical cell containing 5 mL of Britton Robinson pH 7.8 solution. An adequate potential was then applied and once a steady-state current was established, a defined amount of ATI was added to the cell. An oxidation current was observed due to the oxidation of the enzymatic reaction product. Once a steady-state current was set again, set volume of aluminum stock solution were consecutively added and a calibration curve was constructed. As it has been described above, the addition of aluminum solution resulted in a decrease of the chronoamperometric response. Aluminum inhibition effect was quantitatively evaluated by means of the difference between the ATI steady-state current in the absence of aluminum (I_0_) and the steady-state current in the presence of aluminum (I). The parameter ΔI (I_0_- I) was proportional to the amount of ion added. Enzyme electrodes were conditioned in Britton Robinson pH 7 buffer solution for 5 min between each calibration setting.

## 4. Conclusions

The development of a novel biosensor based on the inhibition of acetylcholinesterase using AuNPs/SPCEs allows amperometric determination of aluminum. The biosensor reproducibility and repeatability were studied obtaining values of RSD for the slopes of several calibrations and were lower than 8.1%. The method developed in this work presents several advantages, including lower detection limit, 2.1 μM, than other previous described ones [36].

The easy construction of the biosensors, low cost, disposability, ease-of-use and environmentally friendly features of this method makes it suitable for the analysis of aluminum in water. These characteristics represent clear advantages in comparison to usual analytical methods that allow aluminum determination such as stripping adsorption voltammetry using complexing agents and electrothermic absorption spectroscopy which result tedious and expensive in the routinely analysis of this element.
